# Analyzing asymmetry in brain hierarchies with a linear state-space model of resting-state fMRI data

**DOI:** 10.1162/netn_a_00381

**Published:** 2024-10-01

**Authors:** Danilo Benozzo, Giacomo Baggio, Giorgia Baron, Alessandro Chiuso, Sandro Zampieri, Alessandra Bertoldo

**Affiliations:** Information Engineering Department, University of Padova, Padova, Italy; Padova Neuroscience Center, University of Padova, Padova, Italy

**Keywords:** Differential covariance, Effective connectivity, Brain hierarchy, Time-irreversibility, Resting-state fMRI, Linear state-space stochastic model

## Abstract

This study challenges the traditional focus on zero-lag statistics in resting-state functional magnetic resonance imaging (rsfMRI) research. Instead, it advocates for considering time-lag interactions to unveil the directionality and asymmetries of the brain hierarchy. Effective connectivity (EC), the state matrix in dynamical causal modeling (DCM), is a commonly used metric for studying dynamical properties and causal interactions within a linear state-space system description. Here, we focused on how time-lag statistics are incorporated within the framework of DCM resulting in an asymmetric EC matrix. Our approach involves decomposing the EC matrix, revealing a steady-state differential cross-covariance matrix that is responsible for modeling information flow and introducing time-irreversibility. Specifically, the system’s dynamics, influenced by the off-diagonal part of the differential covariance, exhibit a curl steady-state flow component that breaks detailed balance and diverges the dynamics from equilibrium. Our empirical findings indicate that the EC matrix’s outgoing strengths correlate with the flow described by the differential cross covariance, while incoming strengths are primarily driven by zero-lag covariance, emphasizing conditional independence over directionality.

## Author Summary

Modeling large-scale brain dynamics offers insight into the main principles of brain self-organization. In particular, the identification of traces of nonequilibrium steady-state dynamics also at the mascroscale level has been recently linked to the presence of intrinsic brain networks. Quantifying these aspects is generally limited by numerical difficulties. However, for resting-state BOLD data, a linear stochastic state-space model has demonstrated efficacy, simplifying analysis. Specifically, the asymmetric structure of effective connectivity, that is, the state interaction matrix, directly reflects nonequilibrium steady-state dynamics and time-irreversibility. By disentangling this asymmetry, we quantified departure from equilibrium and discerned primary directions of information propagation, identifying brain regions as sources or sinks.

## INTRODUCTION

An excellent framework for comprehending how the brain spontaneously orchestrates its activities is given by whole-brain resting-state recordings acquired through functional magnetic resonance imaging (fMRI). After substantial efforts aimed at characterizing functional connectivity (FC), which measures steady-state similarity between pairs of brain regions, there has been an increasing focus on investigating dynamic properties (Greene, Horien, Barson, Scheinost, & Constable, 2023; Heitmann & Breakspear, 2018; Schirner, Kong, Yeo, Deco, & Ritter, 2022). Multiple perspectives have been utilized to provide insights into this domain. These range from data-driven approaches, which seek to describe the time variability of functional connectivity (referred to as dynamical FC or dFC; Asadi, Olson, & Obradovic, 2021; Gonzalez-Castillo et al., 2023; Peng et al., 2023), to more complex dynamical systems models that aim to replicate the dynamic structure of the state space (Breakspear, 2017; Wang et al., 2019).

Dynamic causal modelling (DCM) is one of the prominent methods used for modeling fMRI data in a top-down approach (Friston, Harrison, & Penny, 2003). Initially, DCM was conceived to model task-based recordings encompassing a restricted number of regions, within a framework of competing hypotheses. However, more recently, DCM has been extended to encompass whole-brain recordings obtained during resting-state conditions (Frässle et al., 2017; Prando et al., 2020). In any scenario, important aspects of DCM include its Bayesian inference framework, the state-space model with linear state equation, and the nonlinear observational equation designed to address the relationship between neural activity and BOLD signal due to hemodynamic coupling (Friston, Mechelli, Turner, & Price, 2000).

Of significance within this context is the state interaction matrix, referred to as effective connectivity (EC), which captures the causal impact that each component of the system has on the behavior of the other components (Bullmore & Sporns, 2009). The linearity of the state space confines the reconstruction of the phase space around an isolated fixed point, resulting in a first-order approximation of the underlying vector field. This property makes the EC correspond to the Jacobian matrix. On one side, this poses a limitation that makes it challenging to fully grasp nonlinear phenomena like metastability and phase transitions (Cocchi, Gollo, Zalesky, & Breakspear, 2017). However, on the flip side, single-fixed-point dynamical models have demonstrated their effectiveness in describing large-scale brain activity (Nozari et al., 2023; Sip et al., 2023; Siu, Müller, Zerbi, Aquino, & Fulcher, 2022). And this serves as a trade-off, rendering an ill-posed problem more manageable.

In this study, we examine the asymmetry of EC, which serves as an indicator of the nonequilibrium steady-state (NESS) regime (Friston et al., 2021), along with its implications from both physical and biological perspectives. Here, the term equilibrium is used in the context of thermodynamics: It implies an equal probability of transitioning between any states of the system on average. Under this assumption, steady-state equilibrium is achieved by gradient descending the potential energy of the system (dissipative flow) toward its minimum, a condition referred to as detailed balance. Conversely, in the case of nonequilibrium steady-state dynamics, detailed balance is not reached, as a nonvanishing rotational component (solenoidal flow) obstructs convergence to equilibrium. This is due to an exchange of matter, energy, or information with the environment. The presence of this permanent curl flow breaks time-reversal symmetry and establishes a time arrow.

The NESS regime is acknowledged as a fundamental aspect of living systems, as it reflects the ongoing effort to maintain survival by resisting equilibrium (Fang, Kruse, Lu, & Wang, 2019). This attribute characterizes a self-organizing system, which is a fundamental prerequisite for biological systems. Neural activity at the microscale level is known to display nonequilibrium dynamics, such as the generation of action potentials. However, it is unclear to what extent these dynamics extend to macroscale processes and their relationship with higher biological functions (Guzmán et al., 2023). Recent studies have reported evidence of a NESS regime in measurements of macroscopic brain activity. Specifically, there is evidence of task dependence in the degree of departure from equilibrium, as well as a relationship with pathological conditions and states of altered consciousness (Deco et al., 2023; Idesis et al., 2023; Kringelbach, Perl, Tagliazucchi, & Deco, 2023; Lynn, Cornblath, Papadopoulos, Bertolero, & Bassett, 2021). In the language of statistical physics, the NESS regime involves a curl steady-state flow on the potential energy of the system that is connected to a nonzero entropy production rate, thus underlining the dynamics’ time-irreversibility (Wang, 2015). Specifically, the notion of directionality emerges inherently from the direction of rotation of the steady-state flow that is decoupled in its solenoidal component, enabling the recognition of brain regions that act as senders or receivers, thus forming a directed hierarchy. Under our modeling hypothesis, the solenoidal component is driven by the off-diagonal part of the differential covariance, which we will refer to as the differential cross covariance (dC-Cov). The differential covariance is essentially a covariance matrix that quantifies how one node influences the time derivative of another (Kwon, Ao, & Thouless, 2005; Lin, Das, Krishnan, Bazhenov, & Sejnowski, 2017).

We consider it important to shed light on this topic for the following reasons: (a) EC has frequently been portrayed as a directed counterpart of FC in a graph-based manner, often overlooking its underlying context in dynamical system theory. (b) Considering the substantial focus on investigating cortical functional hierarchies at the macroscale (Hilgetag & Goulas, 2020; Margulies et al., 2016), the ability to pinpoint the direction through which information traverses such hierarchies would significantly enhance our comprehension of the phenomenon. (c) Despite potential limitations, the linear approximation offers a distinct advantage by enabling the closed-form calculation of the curl steady-state flow, and related metrics, such as the entropy production rate (Gilson, Tagliazucchi, & Cofré, 2023; Xue, 2016).

This work aims to show how the asymmetry of EC, which is inherently linked to a nonzero dC-Cov, is reflected in the neuronal state in terms of power spectral density and time-irreversibility. This will provide a mechanistic justification for the presence of ultraslow fluctuations, which are a characteristic phenomenon in resting-state recordings (Liégeois et al., 2019). The final part of our work will concentrate on interpreting the row and column strengths of the EC matrix. These are commonly used measures to quantify the incoming and outgoing connectivity of each node (Gilson et al., 2020; Razi et al., 2017). We will consider that EC can be decomposed into the product of the differential covariance and the precision matrices, meaning that each row of the differential covariance is stretched toward the principal components of the precision matrix. By this decomposition into zero-lag covariance (the precision matrix) and differential covariance, we will derive how they contribute to defining the row and column strengths of EC. Our empirical findings revealed that EC row strengths are directly related to the corresponding node strengths of the precision matrix, reflecting conditional independence rather than indicating directionality. On the other hand, the column sums of EC are associated with the corresponding column sums of the steady-state dC-Cov, offering insights into directionality. Since this latter shapes the solenoidal steady-state flow that determines the direction of information propagation, our empirical findings suggest that only the column strengths of EC convey a directional interpretation. Specifically, a large positive column sum suggests a dominant source/sender behavior of the node, while a large negative column sum indicates a dominant sink/receiver behavior. We conclude by providing three examples of applying dC-Cov to uncover the underlying directed hierarchy in resting-state fMRI data, using a mouse dataset and two distinct human datasets.

## RESULTS

We applied sparse-DCM at the single-subject level to both mouse and human data (see Dataset section), and identified the corresponding model. We employed both the model and the implementation described in Prando et al. (2020). Initially, we calculated the dC-Cov (*S* matrix since we assumed uncorrelated noise) and focused on its influence on both the power spectra density (psd) and its time-domain counterpart, that is, the autocorrelation, of the neuronal state.

**Figure 1.**
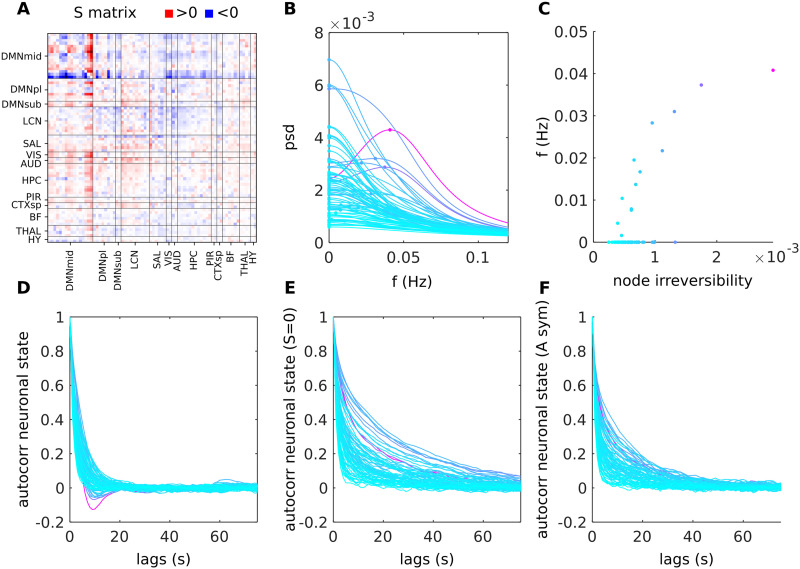
Example of single-subject dC-Cov and its effects on the signal psd and time autocorrelation. (A) The dC-Cov (*S* matrix) for a representative subject from the mouse dataset is displayed. Nodes are categorized based on functional networks. Positive entries are depicted in red, while negative entries are represented in blue. Following the adopted convention, a positive entry indicates that the node in the column acts as the source, while the node in the row serves as the target. (B) The power spectral density (psd) of each neuronal state; color identifies the level of irreversibility of the node from 0 (cyan) to higher values (purple). (C) For each node, how its level of irreversibility relates with the frequency of the psd peak. (D) The autocorrelation of each neuronal state. (E) The autocorrelation of each neuronal state computed by imposing *S* = 0 and deriving the corresponding symmetric *A* matrix. (F) The autocorrelation of each neuronal state inferred from a sparse-DCM model that was constrained to be time-reversible.

A nonzero dC-Cov has a direct impact on the power spectral density of the neuronal state, causing a shift in the frequency peak toward values greater than zero, as depicted in Figure 1B. In other words, a nonzero node irreversibility corresponds to a frequency peak greater than zero in the psd, as shown in Figure 1C (the node irreversibility of node *i* corresponds to the absolute sum of the *i*-th row of the *S* matrix, see Metric Description section). Similarly, in the time-domain, the autocorrelation exhibits a negative peak when nonzero node irreversibility is present, whereas it decreases monotonically, as shown in Figure 1D. When *S* is constrained to be zero either by deriving a new *A* matrix enforcing *S* = 0 from Equation 4 (refer to Figure 1E) or by identifying a time-reversible model (see Figure 1F), the autocorrelation is consistently positive and decreases with frequency. The imposition of time-reversibility results in increasing the memory of the system due to a slower decay in autocorrelation.

To gain a better grasp of how the dC-Cov influences steady-state dynamics, we examined the DCM model under three different situations. These include using the original data and the time-reversed data (i.e., the orginal data time-flipped) with the standard sparse-DCM model, and using the original data with a sparse-DCM designed to maintain symmetry in the *A* matrix, a time-reversible model. The rationale behind time-reversing the original time series and evaluating how the model inference changes aims to test whether the signals contain information on the time directionality. In the case of time-reversible dynamics, we expect no changes in the model after time-reversing the signals, resulting in a zero *S* matrix. However, in the case of time-irreversibility, we expect *S* to become the opposite (each entry changes sign), corresponding to reversing the direction of rotation of the curl steady-state flow.

**Figure 2.**
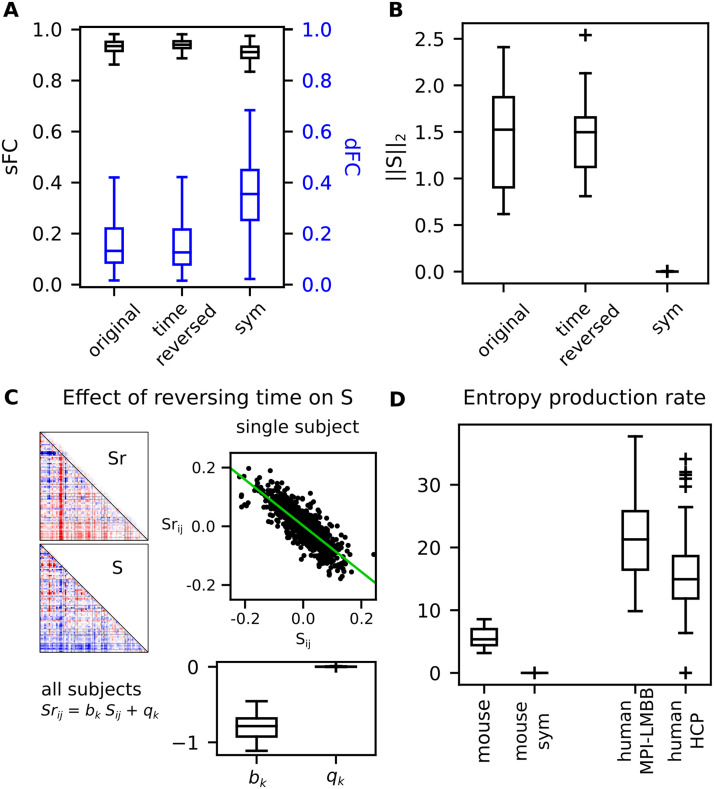
How a nonzero dC-Cov affects the model’s performance and suvsequent entropy production rate. (A) The model’s ability to generate data akin to the empirical mouse recordings is shown in terms of the correlation between their static functional connectivity (sFC) (left y-axis) and the Kolmogorov-Smirnov (KS) distance of their dynamic functional connectivity (dFC) (blue right y-axis). This is demonstrated for the original data, time-reversed data, and the original data fitted with a DCM model designed to maintain symmetry in the effective connectivity (EC). (B) The 2-norm of the *S* matrix is depicted under the three inference conditions: original, time-reversed, and sym (symmetry). (C) The impact of reversing time on *S* is illustrated. For a representative subject, the scatterplot displays the expected negative correlation between corresponding entries of *S* and *S*_*r*_ (the *S* matrix inferred with time-reversal). Additionally, the boxplots show the associated distributions of slope (*b*_*k*_) and intercept (*q*_*k*_) of the single-subject linear fit across the mouse dataset. (D) The entropy production rate computed in the mouse and human datasets.

For the mouse dataset, we initially presented two metrics that quantify the model’s capacity to replicate two key data features (static functional connectivity, sFC, and dynamic functional connectivity, dFC, which are typically measured using the mean correlation matrix; refer to Metric Description section) (see Figure 2A), under the three previously mentioned conditions. This aims to assess the quality of the single-subject model inference by evaluating its capability of generating synthetic fMRI recordings that resemble the sFC and dFC of the empirical one. We observed no substantial distinctions between the inferences made on the original and time-reversed data. It is worth noting that this outcome was anticipated, as the metrics we employed are not sensitive to timedirectionality. Moreover, it is important to mention that the model’s performance declined when symmetry was enforced in the *A* matrix.

In Figure 2B, we reported the 2-norm of the matrix *S* under the three inference conditions. The *S* matrices inferred from the original and time-reversed data demonstrated similar 2-norm values, whereas *S* reduced to zero when symmetry was enforced in the model. These outcomes align with theoretical expectations, which are also reflected in Figure 2C, depicting the impact on the dC-Cov when reversing the time direction of recordings. Each entry of *S* should correspond to its opposite value in *S*_*r*_ (the matrix *S* inferred with time-reversal). In panel C, the scatterplot illustrates the entry-wise relationship between *S* and *S*_*r*_ for a representative subject, while the boxplots display the distributions of slope (*b*_*k*_) and intercept (*q*_*k*_) across the mouse dataset. Finally, in panel (D), we compare the entropy production rates computed as described in Equation 10 for each subject across datasets. Interestingly, both human datasets exhibited higher entropy production rates than the mouse dataset.

**Figure 3.**
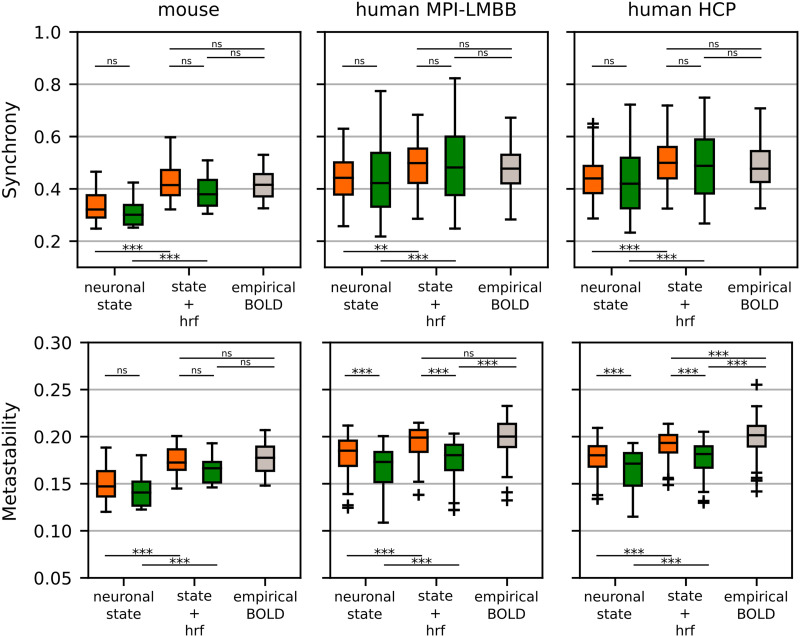
Synchrony and metastability indices in each dataset (mouse, human MPI-LMBB, and human HCP) using both simulated and empirical BOLD signals. When simulating the data, two factors were taken into account: the inclusion of the hemodynamic response function (HRF), labeled in the figure as “neuronal state” vs. “state+hrf,” and the time-reversibility of the model, that is, time-irreversible (orange boxplots) vs. time-reversible (green boxplots). * = *p* < 0.05, ** = *p* < 0.01, *** = *p* < 0.001, and ns = not significant, ANOVA test with Tukey’s multiple comparison test.

After establishing the reliability of our model’s inference in reproducing key FC features and reacting to signal time-reversion, we shifted our focus to examining changes in phase coherence among regions in the simulated data. The presence of alternating periods of coactivation and deactivation among regions is a fundamental characteristic of resting-state fMRI data. These dynamics can be quantified using synchronization and metastability indices, as described in the Metric Description section. These measures stem from the Kuramoto order parameter and were initially developed to investigate phase transitions in models of coupled oscillators (Deco & Kringelbach, 2016; Shanahan, 2010). Given the linear nature of our state model, the phase space possesses only a fixed point, limiting our ability to replicate complex dynamic phenomena. Despite this limitation, we found it valuable to explore eventual disparities between modeled and empirical data in terms of synchronization and metastability. We conducted this analysis using both the original model and its time-reversible version. Moreover, we examined the influence of the hemodynamic function on these measures. It is worth noting that across datasets, data generated from the subject-level sparse-DCM model closely mirrors the synchrony index of the empirical data. This alignment is evident in the top panels of Figure 3, where the orange boxplot in the “state+hrf” columns closely corresponds to the gray boxplot representing the empirical BOLD signal. Likewise, the synchronization index of the time-reversible sparse-DCM (green boxplots) does not exhibit significant differences from the empirical data, indicating that asymmetry may not be necessary to replicate empirical synchronization. The scenario differs when it comes to metastability, where our simulations revealed a significant dependence on the dataset. In the case of mouse recordings, both time-reversible and time-irreversible models managed to replicate metastability, albeit with the time-reversible model (green boxplot) yielding a lower average metastability index. Conversely, for the Max Planck Institute Leipzig Mind - Brain - Body (MPI-LMBB) dataset, only the time-irreversible model effectively reproduced empirical metastability, while neither model could accurately replicate metastability in the Human Connectome Project (HCP) dataset. In all cases, the time-reversible models deviated further from the empirical values. Finally, this analysis sheds light on the role of the hemodynamic response function (HRF) in modeling the BOLD signal. In particular, it highlights its capacity to influence both synchrony and metastability. Indeed, both indices increased if computed on data when the HRF was included.

The second part of the Results section focuses on how a nonzeros *S* defines the directionality on which interactions across regions occur. The research was motivated by a question regarding the interpretability of the strengths of rows and columns in the EC matrix. Specifically, it aimed to determine whether it is valid to interpret the sums of rows and columns in the EC matrix as representing the incoming and outgoing connection strengths, respectively. In this context, our starting point was Equation 4, with a particular focus on the statistical interpretation that each matrix obtained by decomposing *A* has in terms of expectation. To recap, the diagonal matrix Σ_*w*_ contains the variance of the node random fluctuations (assumed constant across nodes), Σ represents the steady-state covariance matrix, denoted as 𝔼[*x*(*t*)*x*(*t*)^⊤^]|_*t*→∞_, and its inverse Σ^−1^ is the precision matrix. This latter holds a direct interpretation in terms of conditional independence: A value of zero or very close to zero indicates that the two signals are conditionally independent given the rest of the recordings (Das et al., 2017). Finally, the steady-state dC-Cov, *S* with off-diagonal entries *S*_*i*,*j*_ = 𝔼[$\dot{x}$_*i*_(*t*)*x*_*j*_(*t*)^⊤^]|_*t*→∞_ and zero diagonal, which facilitates the identification of source and sink nodes in each pair. Note that dC-Cov and *S* coincide only under the assumption of uncorrelated noise, otherwise dC-Cov will also be influenced by the presence of correlated noise.

Beginning with the EC row strengths, it has been observed from Equation 7 that the sum of its *i*th row can be decomposed as a linear combination. This combination involves regressors that are equal to the row sums of the precision matrix Σ^−1^ and coefficients that are the elements in the *i*th row of *S*. Additionally, there is the term $\Sigma_{i,.}^{-1}$ weighted by −*σ*^2^/2, which accounts for the dissipative contribution that is mediated by the dA-Cov.

**Figure 4.**
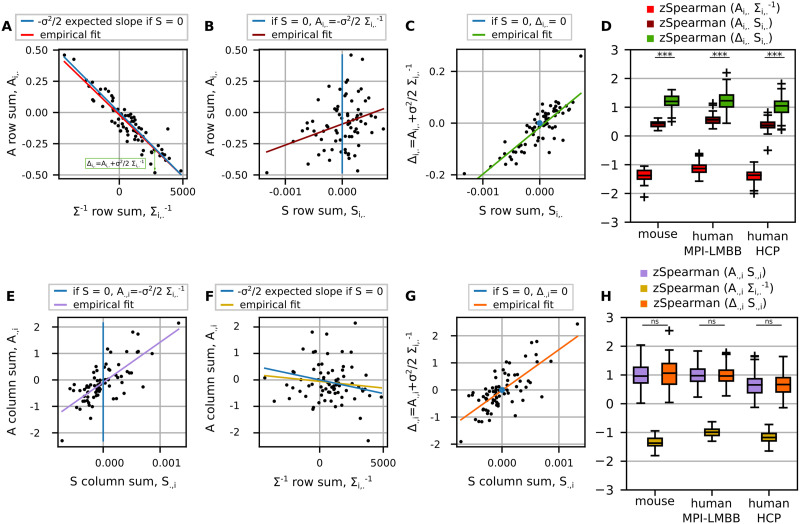
How the row and column strengths of the EC matrix (*A*) relate to the corresponding strengths computed on the matrices on which *A* can be decomposed, that is, Σ^−1^ and *S*. Panels on the top refer to the EC row strengths: In panel A the relationship between the row sums of *A* and the corresponding row sum of Σ^−1^ (the regressors in Equation 7); in panel B how *A* row strengths relate to the row sums of *S* (the coefficients in Equation 7), in panel C *S* row strengths are shown in relation to Δ_*i*,._ which isolate the contribution of *S* in the row strength of *A*; and panel D summarizes the previous relationships by showing the distributions of the Fisher-transformed Spearman correlations across subjects for each dataset. Statistical *t* test was performed to compare the use of *A* or Δ to reveal what encoded in the row sum of *S*. Δ performed significantly better, *p* < 0.001. Panels on the bottom refer to the EC column strengths: in panel E the relationship between the column sums of *A* and the corresponding column sums of *S* (the regressors in Equation 8); in panel F how *A* column strengths relate to the node strengths of Σ^−1^ (the coefficients in Equation 8), and panel G the column sum of Δ against the one of *S*. Panel H generalizes the previous results across subjects and datasets, similarly to panel D. No significant differences between using *A* or Δ.

In Figure 4, the first row illustrates how *A* row sums relate with Σ^−1^ row sums (panel A) and with *S* row sums (panel B). In the case of the former, a strong negative correlation is evident. However, in the latter, there is no clear pattern. This suggests that in our resting-state BOLD recordings, even in the presence of a nonzero *S* matrix, the relationship between row sums of *A* and Σ^−1^ is primarily preserved. It is important to note that in the case of a time-reversible model, there would be a perfect linear relationship with a slope equal to −*σ*^2^/2, as indicated by the blue line in panel A, while the influence of the dC-Cov does not emerge from the row sums of *A*.

To emphasize the effect of the dC-Cov, we need to isolate its contribution to the row sums of *A*, which shows how much each row sum deviates from the time-reversible condition. In Figure 4A, this is represented in green as Δ_*i*,._ = *A*_*i*,._ + *σ*^2^/2$\Sigma_{i,.}^{-1}$, and is shown in a scatterplot against the row sum of *S* in panel C. Now, the contribution of the solenoidal flow becomes apparent as these two variables linearly correlate. Note that Δ = *S*Σ^−1^ represents the gradient of the solenoidal flow.

The scatterplots in Figure 4 pertain to a representative mouse subject. Comprehensive results across datasets are depicted in panels D and H.

Regarding the EC column strengths, as reported in Equation 8 for each node *i*, its column sum is a linear combination with regressors the row sums of *S* and coefficients the *i*th column of Σ^−1^, plus the term in Equation 6. Figure 4E illustrates the relationship between the column sums of *A* and the corresponding column sums of *S*. It appears that the column sums of *A* positively correlate with the corresponding column sums of *S*, indicating that the solenoidal part is reflected in the columns of *A*. In panel F, we also explored the relationship between the column sums of *A* and the corresponding row sums of Σ^−1^. The expected relationship for a time-reversible model is represented by the blue line, but the empirical values do not reveal any significant trend between them. For the sake of completeness, in panel G, we examined the relationship between the column sums of Δ and the column sums of *S*. Global findings presented in panel H validate that the influence of the dC-Cov at the node level can be inferred from the columns of the EC. This observation holds true regradless of whether the dissipative component is considered, as there were no significant differences between using *A* or Δ.

**Figure 5.**
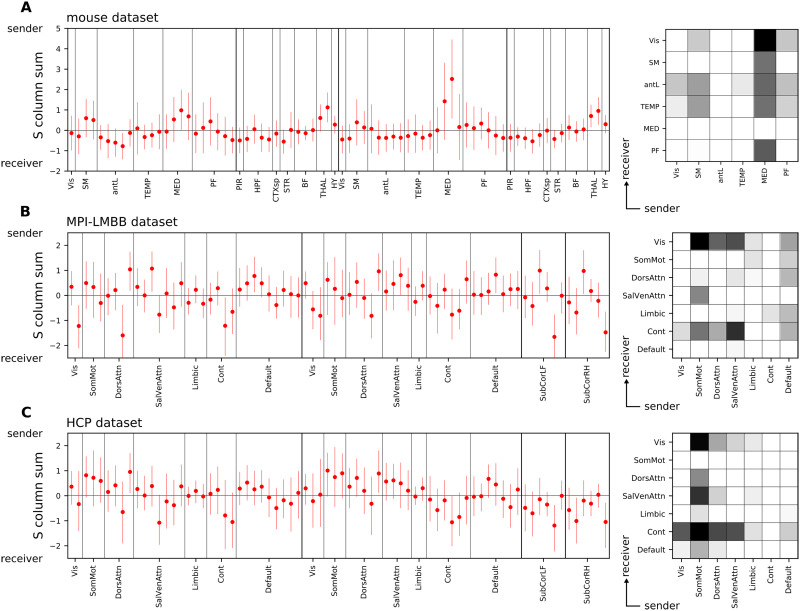
The average in/out node profile and in/out network matrix across subjects for each dataset are presented in the following panels. In the in/out network matrix, only positive values are displayed, with the grayscale color bar transitioning from lighter to darker shades of gray to indicate that the network on the x-axis predominantly interacts as a sender with the network on the y-axis. In panel A, the in/out node profile and network matrix are derived from the mouse data. Panels B and C display the in/out node profiles and network matrices of the MPI-LMBB and HCP datasets, respectively. For each node, a negative column sum in the matrix *S* indicates a prevalence of the node behaving as a receiver, while a positive column sum in *S* suggests a prevalence of the node behaving as a sender.

We conclude the Results section by showing the column sums of *S* in each dataset, the in/out node profile, and a reduced version of dC-Cov in which entries referring to the same pair of networks have been averaged (in/out network matrix), see Figure 5. Considering the interpretation of the dC-Cov, its column strengths quantify the prevalence of a node acting as a source (positive strength) or a sink (negative strength) within the system. In panel (a), we present the in/out node profile and the in/out network matrix derived from the mouse dataset. Similarly in panels (b) and (c) for the MPI-LMBB and HCP datasets, respectively. The correlation coefficient between the two human profiles is 0.73 (*p* < 0.001) and between the in/out network matrices is 0.89 (*p* < 0.001), suggesting good reproducibility across datasets and enhancing the reliability of our results. In Seguin, Razi, and Zalesky (2019) a similar analysis was carried out using structural connectivity (SC) data. In the case of human data, they utilized undirected SC based on diffusion MRI, and for the mouse case, directed SC obtained from the connectome in Oh et al. (2014). The study focused on employing asymmetric network communication measures with the objective of identifying sender and receiver nodes, with which we compared our findings. In particular, the strongest correlations with our results were obtained when comparing with the asymmetric measure based on diffusion efficiency. The in/out network matrices in Figure 5 exhibited Pearson correlation coefficients of 0.55 (*p* = 0.03) for the mouse dataset, 0.57 (*p* < 0.01) for the MPI-LMBB dataset, and 0.52 (*p* = 0.02) for the HCP dataset. Supporting Information Figure S1 displays the coupling at the single-subject level with the diffusion efficiency asymmetry, across different SC densities, for each functional dataset. The couplings with diffusion efficiency were computed using both EC − EC^⊤^ and dC-Cov. A consistently stronger coupling with structural asymmetry was observed when using dC-Cov.

## DISCUSSION

In this work, we explored the implications of having a nonzero differential cross-covariance (dC-Cov) in the modeling of fMRI data using a linear state-space stochastic model. Specifically, we examine the DCM-fMRI framework, which incorporates an observational equation to model the hemodynamic transfer function, that is, the forward model. DCM, initially employed for task-based recordings and more recently for resting-state data, is a well-established family of top-down models. In this framework, the state interaction matrix is referred to as the effective connectivity (EC) matrix. This matrix is known for its ability to convey directional and causal information through its asymmetric structure. This stands in contrast to functional connectivity (FC), which is generally symmetric. Consequently, EC has frequently been employed to differentiate between incoming and outgoing node connections. Indeed, while the asymmetry of EC is not a novel concept, there has been a relative lack of attention directed toward elucidating the underlying reasons for its asymmetric nature and the mechanistic implications when generating surrogate data utilizing a state matrix of such a form.

The asymmetry of EC is a consequence of a nonzero dC-Cov, and this is intricately linked with the presence of non-flat brain hierarchies. Therefore, the central question of this work focuses on how we can discern a non-flat brain hierarchy from the data, identify the patterns of brain regions involved, and ascertain the direction in which the hierarchy functions.

We showed that the crucial element for addressing these questions is the *S* matrix that coincides with the steady-state dC-Cov in our formulation. We derived it at the single-subject level by identifying a stochastic DCM model using an inference method known as sparse-DCM (Prando et al., 2020). In particular, our study utilized a matrix decomposition that enables the separation of the zero-lag statistic from the nonzero-lag statistic, and highlighted the role of the latter component, which imparts the asymmetric shape to the state matrix. Interestingly, when considering a stochastic model near a stable fixed point with Gaussian fluctuations (Kwon et al., 2005), this decomposition can be interpreted from the perspective of statistical physics as representing the splitting of the nonequilibrium steady-state dynamics into two orthogonal components: the gradient of the potential energy (a dissipative gradient flow) and the curl steady-state flow (a solenoidal flow; Friston et al., 2021). The curl steady-state flow, which in our modeling framework is mediated by the dC-Cov, is a manifestation of a nonequilibrium dynamics. Nonequilibrium dynamics have been found to be ubiquitous among living systems (Gnesotto, Mura, Gladrow, & Broedersz, 2018), and they also manifest within resting-state functional magnetic resonance imaging (rsfMRI) recordings (Krakauer, 2023).

A nonzero dC-Cov has several effects on the data. First, it underscores the significance of time-delayed interactions, which have been consistently highlighted as playing a central role in rsfMRI data. For example, in Shinn et al. (2023), it has been shown how both time and spatial autocorrelation can account for a significant portion of the network topological measures commonly used in connectome analyses. In Castaldo et al. (2023), the significance of delayed interactions has been highlighted when modeling neural data. Additionally, in Bolt et al. (2022), the connection between various empirical phenomena observed in large-scale brain organization and both zero-lag and time-lag statistics has been explored. Moreover, time-delayed interactions define the so-called arrow-of-time (Bolton, Van De Ville, Amico, Preti, & Liégeois, 2023; Deco et al., 2023), which is inherently linked to the concept of entropy production rate, the center of recent works, such as Kringelbach et al. (2023) and Lynn et al. (2021).

Interestingly, as previously reported in the literature, time-irreversible dynamics can give rise to slow fluctuations owing to the presence of oscillatory modes represented by the complex eigenvalues of the Jacobian matrix. It is reasonable to assume that these oscillatory features, spanning various timescales and spatial patterns, are linked to or can be considered to be linear projections (in the case of nonlinearity) of phenomena such as harmonic waves (Atasoy, Donnelly, & Pearson, 2016), traveling waves (Bolt et al., 2022), oscillatory modes (Cabral et al., 2022), brain spirals (Xu, Long, Feng, & Gong, 2023), and cascade propagation (Rabuffo, Fousek, Bernard, & Jirsa, 2021).

Examining the contribution of each brain region to the definition of the curl steady-state flow reveals its role in shaping how information propagates throughout the brain hierarchy and its overall tendency to primarily function as a sender or a receiver. The notion of brain hierarchy is widely recognized in systems neuroscience, as highlighted in Hilgetag and Goulas (2020). Of interest for this discussion is the functional cortical hierarchy, which reflects graph structural properties of the functional connectivity matrix (Margulies et al., 2016). In recent years, significant progress has been made in understanding functional connectomics, including the identification of major functional gradients that explain key aspects of functional connectivity. However, there has been a gap in understanding the directional aspects of information propagation along these gradients.

Alongside these theoretical considerations, a pivotal role is played by the model identification algorithm used to estimate the parameters of the DCM model. In our study, we employed a recently developed approach to address the inference problem in the case of rsfMRI. This approach incorporates two critical components: a sparsity-inducing prior applied to the matrix EC and a linearized region-specific hemodynamic response function (HRF; Prando et al., 2020). Part of our results, particularly those presented in Figures 2 and 3, were intended to provide evidence regarding the model identification process. We focused on standard metrics commonly used in the literature for this purpose. They include the model’s ability to replicate the empirical static FC and the range over which dynamic FC values are distributed. Collectively, this comparison demonstrated that the inferred models generally exhibited good generalization properties, except for the time-reversible model. This finding supports the idea that a nonequilibrium dynamics model provides a better explanation for rsfMRI data.

We also evaluated other metrics, specifically the synchrony and metastability indices. Although these metrics may seem at odds with the stable single fixed point around which the dynamics were modeled, as they are associated with nonlinear phenomena, we deemed it important to assess the disparities between surrogate and empirical data. Notably, only the metastability of the HCP dataset was not replicated by the surrogate data, while in all other cases, the simulated data exhibited indices that were not significantly different from the empirical ones.

We should examine this evidence from two perspectives. On one side, it is important to consider the specific characteristics of each dataset, such as the use of halothane anesthetic in the mouse data, variations in sampling rates across datasets, and notably, the fact that the HCP dataset has the lowest sampling rate. On the other side, we should also acknowledge that recent research has reported the ability of a linear state-space model to replicate large-scale brain dynamics successfully (Nozari et al., 2023; Ponce-Alvarez & Deco, 2024; Sip et al., 2023).

In this context, it is worth mentioning the advantages offered by the linear state-space model when computing the steady-state dC-Cov and metrics related to time-irreversibility, such as the entropy production rate, for which closed-form solutions are available (Gilson et al., 2023). Alternative approaches have been proposed and tested for estimating dC-Cov in previous researches, for example, Chen, Bukhari, Lin, and Sejnowski (2022), Kim et al. (2023), and Lin et al. (2017). Additionally, in Lynn et al. (2021), a hierarchical clustering algorithm was used to sample the system phase space and derive the entropy production rate, while Deco, Perl, Bocaccio, Tagliazucchi, and Kringelbach (2022) introduced an estimator of time-irreversibility by comparing the correlation between forward and backward signal lag-correlations.

The second part of our work delved into the issue of interpretation. Specifically, we explored whether the EC matrix or the steady-state dC-Cov offers a more appropriate description of how information flows within the brain hierarchy.

Traditionally, the EC matrix has been used to reveal directed connections within the brain (Entz et al., 2014). However, when we consider the role of EC as a state-space matrix, it provides timing information by modeling the trajectory of the state space for each initial condition. In doing so this matrix combines various data statistics, including the variance of noise fluctuations, the zero-lag covariance, and the dC-Cov, as seen in Equation 4. On the other hand, the steady-state dC-Cov serves a distinct purpose. First, it isolates the causal component of the EC (what makes EC asymmetric). Additionally, it acts as rotation matrix so it mediates the solenoidal component of the system’s dynamics (what contributes to making the hierarchy non-flat). Note that it is not enough to separate the asymmetric part of EC since this could be done also by computing EC − EC^⊤^ (Friston, Li, Daunizeau, & Stephan, 2011); however, this separation of the asymmetric part does not represent the steady-state dC-Cov.

In accordance with the conventional definition of EC incoming node strength, our empirical observations highlighted the substantial impact of the precision matrix in estimating a node’s incoming strength, while the influence of the dC-Cov became evident primarily after the dominant contribution of the precision matrix had been factored out. Conversely, when calculating the outgoing node strength from EC, a consistent linear relationship with the dC-Cov was maintained—thus suggesting for this latter a coherent interpretation in terms of directionality.

Building upon the interpretation of the dC-Cov, we devised a straightforward metric to encapsulate each node’s overall role within the network. This metric involved summing the columns of the dC-Cov matrix for each node, obtaining what we called in the Results section the average in/out node profile. A positive sum indicated that the node primarily acted as a source, whereas a negative sum suggested a sink role. It is important to note that by computing the row sums and reversing the interpretation, we would arrive at the same conclusions. Likewise, instead of reducing the role of each node to a single number, which would overlook network heterogeneity, we also presented the in/out network matrix. In this matrix, entries of the dC-Cov matrix were averaged, providing insight into how each pair of networks interacts in terms of source or sink behavior.

Concerning the mouse dataset, a predominant sender role was assigned to the medial (MED), prefrontal (PF), and somatomotor (SM) regions that constitute the DMNmid and LCN functional networks. This aligns with findings from Coletta et al. (2020), where these regions exhibited a high out/in structural connection ratio, while a receiver role at the cortex level is mainly played by anterolateral (antL) areas, of which its main component is the agranular insular area that forms the salience network. Additionally, our results revealed the thalamic and hypothalamic regions as prominent senders, even though a specific preferred directionality was not previously reported in the cited work but however identified as global connector hubs. As main subcortical receivers, we identified the hippocampus and the dorsal striatum (DMN subnetwork), which was reported as main receiver also in Coletta et al. (2020) being part of the basal ganglia.

In relation to the human datasets, we first highlight the high degree of consistency between the corresponding results. This consistency may be attributed in part to the use of the same parcellation scheme. Nevertheless, it adds robustness to our findings, as it indicates that similar results were obtained from two separate independent datasets. Additionally, our results align with Seguin et al. (2019), which also explored the asymmetric behavior of brain regions in terms of being a sink or source using network communication measures on structural imaging data (diffusion MRI). Specifically, our results support the concept that information travels through the functional hierarchy, progressing from sensory/motor and unimodal regions to integrative multimodal ones responsible for higher order functions. This explains the dominant sender activity of the somatomotor network, primarily communicating with the visual, ventral attention, and control networks, and similarly, the connections from the ventral attention network to the visual and control networks, as well as the role of the control network as a primary receiver from the other networks. The widespread agreement in the literature regarding this directional arrow is substantiated by evidence from various sources. These include studies on structural data (Markov et al., 2013; Seguin et al., 2019; Tanner et al., 2022), investigations into functional gradients (Margulies et al., 2016), and dynamic models (Deco, Vidaurre, & Kringelbach, 2021; Demirtaş et al., 2019; Parkes et al., 2022). According to this body of work, the default mode network should occupy the apex of the hierarchy and primarily receive inputs from all other networks. However, our findings reveal a different pattern. In the MPI-LMBB dataset, the default mode network takes on a sender role, while in the HCP dataset, it does not seem to exhibit a distinct preferred behavior. This observation is in concordance with what is hypothesized by the predictive processing framework. This framework postulates the existence of two counteracting information flows within the brain: top-down prediction signals (feedback signals) and bottom-up prediction errors (feedforward signals; Chen, Henson, Stephan, Kilner, & Friston, 2009; Katsumi, Theriault, Quigley, & Barrett, 2022). It is plausible that the dominant behavior of the default mode network in our results can be attributed to the prevalence of top-down prediction signals originating from it.

A similar analysis based on the asymmetry of EC was conducted also in Seguin et al. (2019). In their analysis, asymmetry was computed by subtracting EC from its transpose. However, as previously mentioned, this approach captures the asymmetric part of EC but does not represent the dC-Cov. In their comparison using a cortical partition into seven networks (the same partition used in our analysis), they did not observe a significant correlation between the asymmetry derived from EC and the asymmetry derived from communication-based measures. Similarly, our dC-Cov showed no significant correlation with the EC asymmetry reported in Seguin et al. (2019), whereas it did correlate with the asymmetry derived from diffusion MRI data.

Future research will delve deeper into the exploration of the steady-state dC-Cov within the framework we have presented here. One crucial aspect, which we have yet to explore in this study but believe holds significant importance, is the differentiation of dC-Cov across various timescales. It might be overly simplistic to assume that the dynamics of large-scale brain activity are governed by a single hierarchy. Evidence in favor of this notion can be found in Sorrentino et al. (2023), which elucidates the interaction between top-down and bottom-up processes. Additionally, in Brown, Lee, Pasquini, and Seeley (2022), authors demonstrated the intricate interaction between gradient-specific dynamics and dynamics across gradients. And, in Chen et al. (2023), researchers leveraged eigen-microstate analysis to identify basic modes in rsfMRI data. However, in most of these examples, a low-dimensional representation of brain activity was found, and each dimension characterized by its specific pattern of interactions, but what is missing is the consideration of directionality. This could be studied as a continuation of this work by applying an eigen-mode decomposition to the effective connectivity matrix and projecting the dC-Cov onto each mode. This could help disentangle the brain hierarchy across different timescales.

Another limiting aspect of our analysis is that we mainly presented results at the population level. This is a point that we will carefully consider in future work. Recent studies have demonstrated the relationship between the level of asymmetry in brain hierarchy and brain states evoked by task conditions (Lynn et al., 2021), levels of conscious awareness (Perl et al., 2021), neuropsychiatric diseases (Deco et al., 2023), and the presence of stroke lesions (Idesis et al., 2023). This suggests the potentiality of deriving subject-specific biomarkers based on properties of brain hierarchies.

## MATERIALS AND METHODS

### Dataset

#### Mouse dataset.

A dataset of *n* = 20 adult male C57BI6/J mice were previously acquired at the IIT laboratory (Italy). All in vivo experiments were conducted in accordance with Italian law (DL 2006/2014, EU 63/2010, Ministero della Sanità, Roma) and the recommendations in the Guide for the Care and Use of Laboratory Animals of the National Institutes of Health. Animal research protocols were reviewed and consented to by the animal care committee of the Italian Institute of Technology and Italian Ministry of Health. Animal preparation, image data acquisition, and image data preprocessing for rsfMRI data have been described in greater detail elsewhere (Gutierrez-Barragan, Basson, Panzeri, & Gozzi, 2019). Briefly, rsfMRI data were acquired on a 7.0-T scanner (Bruker BioSpin, Ettlingen) equipped with BGA-9 gradient set, using a 72 mm birdcage transmit coil, and a four-channel solenoid coil for signal reception. Single-shot BOLD echo planar imaging time series were acquired using an echo planar imaging sequence with the following parameters: repetition time/echo time, 1,000/15 ms; flip angle, 30°; matrix, 100 × 100; field of view, 2.3 × 2.3 cm^2^; 18 coronal slices; slice thickness, 0.60 mm; 1,920 volumes.

Regarding image preprocessing as described in Gutierrez-Barragan et al. (2022), time series were despiked, motion corrected, skull stripped, and spatially registered to an in-house EPI-based mouse brain template. Denoising and motion correction strategies involved the regression of mean ventricular signal plus six motion parameters (Rocchi et al., 2022). The resulting time series were then band-pass filtered (0.01–0.1 Hz band). After preprocessing, mean regional time series were extracted for 74 (37 + 37) regions of interest (ROIs) derived from a predefined anatomical parcellation of the Allen Brain Institute (Oh et al., 2014; Wang et al., 2020).

#### MPI-LMBB dataset.

A subset of 295 subjects has been selected from the resting-state scans of the publicly available MPI-Leipzig Mind-Brain-Body dataset (https://legacy.openfmri.org/dataset/ds000221/). The data selection was performed on the original dataset (consisting of 318 individuals) by excluding participants with high motion or preprecessing failures. Then, half of the dataset was utilized for clustering purposes (detailed in the following section). From the remaining portion, we selected only younger participants aged between 20 and 30 years, resulting in a final sample of 107 subjects.

Data acquisition was performed with a 3T Siemens Magnetom Verio scanner, equipped with a 32-channel head coil. The protocol included a T1-weighted 3D magnetization-prepared 2 rapid acquisition gradient echoes (3D-MP2RAGE), and resting-state fMRI images were acquired using a 2D multiband gradient-recalled echo EPI sequence (TR = 1,400 ms; flip angle = 69°; voxel size = 2.3 × 2.3 × 2.3mm; volumes = 657; multiband factor = 4) and two spin echo acquisitions. For additional information regarding the acquisition protocol, please refer to Babayan et al. (2019). Details concerning the preprocessing of rsfMRI data can be found in Silvestri et al. (2022). Subsequently, the temporal traces were subjected to band-pass filtering in the frequency range of 0.0078 to 0.1 Hz and temporally despiked by means of the *icatb_despike_tc* function of the GIFT toolbox (https://trendscenter.org/software/gift/).

#### HCP Young Adult dataset.

We used the HCP Young Adult (HCP-YA) data release, which included 1,200 normal young adults, aged 22–35 (Van Essen et al., 2012). All data were collected on a 3T Siemens Skyra scanner with gradients customized for the HCP. We restricted our analysis to 173 subjects (age range 22–35 years). Resting-state rfMRI data were acquired in four runs of approximately 15 each, two runs in one session and two in another session, with eyes open with relaxed fixation (sequence parameters for each run: gradient-echo EPI, TR = 720 ms, TE = 33.1 ms, flip angle = 52°, FOV = 208 × 180, voxel size = 2 mm isotropic, frames = 1,200). Within each session, oblique axial acquisitions alternated between phase encoding in a right-to-left (RL) direction in one run and phase encoding in a left-to-right (LR) direction in the other run (Van Essen et al., 2012).

Concerning data preprocessing, the dataset used in this study was the preprocessed volumetric version provided by HCP (Glasser et al., 2013), which combines a set of tools from FSL, FreeSurfer, and the HCP Connectome Workbench (Marcus et al., 2013). It encompasses three MR structural pipelines for distortion correction and the alignment of individual brain data to a standardized MNI template. The fMRI data were preprocessed using the MR functional pipeline, which involved distortion correction, motion correction by aligning fMRI volumes, and registration of the fMRI data to the corresponding structural data. For each subject, only the second run (Run 2) of the data was utilized. Additionally, the data were temporally filtered with a band-pass range of 0.0078–0.1 Hz and temporally despiked by means of the *icatb_despike_tc* function of the GIFT toolbox.

### Cortical Network Partitions

In the mouse data, we partitioned the cortical areas into six macro-regions (Harris et al., 2019). These regions are: the somatomotor area (SM), which corresponds to the functional lateral cortical network (Liska, Galbusera, Schwarz, & Gozzi, 2015); the visual area (Vis); the temporal area (TEMP); the anterolateral area (antL), which mainly overlaps with the salience network (agranular insula areas) and part of the default mode posterolatera network (DMNpostl); the medial area (MED) representing part of the default mode midline network (DMNmid) (Gutierrez-Barragan et al., 2022); and the prefrontal area (PF), which completes the anterior part of DMNmid. In terms of subcortical regions, our adopted parcellation includes the hippocampus (HP), the striatum regions (STR), the basal forebrain (comprising the lateral septal complex, striatum-like amygdalar nuclei, and pallidum), the thalamus, and the hypothalamus. For a comprehensive list, please refer to Supporting Information Table S1.

Regarding the human datasets, in both cases we extracted time series data from a 100-area parcellation scheme of the cortex provided by the Schaefer atlas (Schaefer et al., 2018), which maps to seven resting-state functional networks referring to both hemispheres: visual network (Vis) (5 parcels), somatomotor network (SomMot) (6 parcels), dorsal attention network (DorsAttn) (9 parcels), saliency/ventral attention network (SalVenAttn) (11 parcels), limbic network (Limbic) (5 parcels), control network (Cont) (10 parcels), and default mode network (DMN/default) (16 parcels). We also defined a set of 12 subcortical regions based on the AAL2 segmentation (Rolls, Joliot, & Tzourio-Mazoyer, 2015). For each hemisphere, we selected six regions consisting of thalamus proper, caudate, putamen, pallidum, hippocampus, and cerebellum.

A consensus clustering procedure was applied to reduce the number of cortical parcels to reach a parcellation compatible with the DCM inference algorithm. As a result, we applied to the first half of the MPI-LMBB dataset (147 subjects) a consensus clustering evidence accumulation (CC-EA) procedure, similarly to that described in Ryali, Chen, Padmanabhan, Cai, and Menon (2015), to determine the number of optimal clusters. Specifically, this framework employs base and consensus clustering methods to get robust and stable clusters. Details on the procedure can be found in Baron, Silvestri, Benozzo, Chiuso, and Bertoldo (2023). The clustering procedure yielded 62 cortical clusters that, when combined with the 12 subcortical regions, resulted in a total of 74 parcels. For a comprehensive list, please refer to Supporting Information Table S2.

### State-Space Linear Model

The linear DCM framework employs a linear stochastic model to describe the neuronal state. This model represents a continuous-time linear time-invariant stochastic system:

$$\dot{x}\left(t\right)=Ax\left(t\right)+w\left(t\right),$$ (1)

where *x* ∈ ℝ^*n*^ is the vector containing the states of the network nodes, *A* ∈ ℝ^*n*×*n*^ is the state interaction matrix (the so-called effective connectivity matrix, EC), and the input *w* is a zero-mean white noise vector with positive definite covariance matrix Σ_*w*_ = 𝔼[*w*(*t*)*w*(*t*)^⊤^]. We assumed uncorrelated noise across nodes so that Σ_*w*_ = *σ*^2^*I*_*n*_, where *σ*^2^ represents the variance of each brain endogenous fluctuation and *I*_*n*_ the identity matrix of size *n*.

Moreover, *A* is assumed to be stable (its eigenvalues have strictly negative real part); this ensures the existence of a positively definite steady-state covariance matrix Σ, which, after normalization, represents the functional connectivity matrix (FC):

$$\Sigma ≔\lim_{t\to \infty }𝔼\left[x\left(t\right)x{\left(t\right)}^{⊤}\right],$$ (2)

for which the algebraic Lyapunov equation holds:

$$A\Sigma +\Sigma A^{⊤}+\Sigma_{w}=0.$$ (3)

Importantly, *A* can be decomposed such that

$$A=\left(-\frac{1}{2}\Sigma_{w}+S\right)\Sigma^{-1}.$$ (4)

Equation 4 parameterizes all *A* matrices that give the same Σ, that is, the covariance matrix of the state, for any *S* skew-symmetric matrix, that is, *S* = −*S*^⊤^. This defines a one-to-one map between each (Σ, *S*) and *A* given the noise covariance Σ_*w*_ (Casti et al., 2023), with *S* ≔ $\frac{1}{2}$(*A*Σ − Σ*A*^⊤^). Any off-diagonal element (*i*, *j*) of *S* can be written in statistical term as lim_*t*→∞_ 𝔼[$\dot{x}$_*i*_(*t*)*x*_*j*_(*t*)^⊤^], and we refer to this matrix as the steady-state differential cross-covariance (dC-Cov). Similarly, the differential auto-covariance (dA-Cov) is represented by −Σ_*w*_/2. The sum of these two matrices constitutes the differential covariance matrix (Lin et al., 2017). The equivalence between the off-diagonal entries of the differential covariance and *S* arises from assuming Σ_*w*_ to be a diagonal matrix, that is, uncorrelated noise. However, if correlated noise is present, as not considered in this study, dC-Cov and *S* are no longer interchangeable.

Equation 4 can also be seen as following from the fundamental theorem of vector calculus (or Helmholtz decomposition), which states that any vector field in steady state can be broken down into the combination of a dissipative (curl-free) flow that is mediated by −Σ_*w*_/2 (dA-Cov), and a solenoidal (divergence-free) flow that is mediated by *S* (dC-Cov) (Friston et al., 2021; Zia & Schmittmann, 2007). The existence of both of these flows constitutes the core of nonequilibrium steady-state dynamics. A nonzero *S* matrix introduces time-irreversibility into the dynamics, and inverting the sign of *S* results in the reversal of dynamics (Costa, Friston, Heins, & Pavliotis, 2021).

The differential cross-covariance matrix *S* gives a direct interpretation of which node behaves as source and which as sink (Chen et al., 2022; Lin et al., 2017). If *S*_*i*,*j*_ > 0, then node *j* is considered the source and node *i* is considered the sink, indicating that information is flowing from node *j* to node *i*. In the context of *S* being skew-symmetric, this example implies *S*_*j*,*i*_ < 0. Therefore, to maintain coherence with the established interpretation, the row entry corresponds to the source and the column entry corresponds to the sink.

This interpretation provides a straightforward method to quantify the prevalence of a node acting as a source or sink within the system. For example, summing the values in each column of the *S* matrix allows us to gauge the extent to which a node predominantly serves as a source (if the sum is largely positive), serves as a sink (if the sum is largely negative), or maintains balance between the two roles (if the sum is close to zero). A sum of zero may also indicate that the node is excluded from contributing to the steady-state solenoidal flow.

In characterizing nodes within a network, matrix *A*, representing effective connectivity, is commonly employed to quantify incoming and outgoing strengths. Typically, for a given node, its incoming and outgoing strengths are calculated as the sums of the node’s corresponding row and column in matrix *A*, respectively. In light of our previous part about the pivotal role of *S* in shaping the directionality of steady-state dynamics, our objective is to study how this is reflected on the asymmetry of *A*. In the following, we will use the notation *A*_*i*,._ and *A*_.,*i*_ to represent the row and column sum of node *i* in *A*, respectively. If *A* is symmetric the following holds:

$$A=-\frac{\sigma^{2}}{2}\Sigma^{-1}.$$ (5)

Thus, *A* is the outcome of the multiplication of the dA-Cov by the precision matrix, yielding a scaled version of the precision matrix. Since the precision matrix is symmetric and the noise variance is assumed equal across regions, incoming and outgoing strengths of any node *i* are equal:

$$A_{i,.}=A_{.,i}=-\frac{\sigma^{2}}{2}\Sigma_{i,.}^{-1},$$ (6)

while in the presence of causal interactions among regions, which corresponds to a nonzero dC-Cov, that is, *S* ≠ 0, from Equation 4 it follows that the incoming strength of node *i* becomes the following:

$$A_{i,.}=\sum_{j=1}^{n}S_{i,j}\Sigma_{j,.}^{-1}-\frac{\sigma^{2}}{2}\Sigma_{i,.}^{-1},$$ (7)

that is, a linear combination computed by taking the sum of rows (or columns) of the precision matrix, that is, $\Sigma_{i,.}^{-1}$, where each row sum (or column sum) is weighted by the corresponding element in the *i*th row of matrix *S*, plus the term in Equation 6. Similarly, the outgoing strength of node *i* becomes

$$A_{.,i}=\sum_{j=1}^{n}\Sigma_{j,i}^{-1}S_{.,j}-\frac{\sigma^{2}}{2}\Sigma_{.,i}^{-1},$$ (8)

thus a linear combination where each *S* column sum is weighted by the corresponding element in the *i*th column of the precision matrix, plus −$\frac{\sigma^{2}}{2}\Sigma_{.,i}^{-1}$, that is, the strength in the symmetric case (Equation 6).

### Sparse-DCM

The effective connectivity matrix, along with other model variables, was estimated at the single-subject level using the method described in Prando et al. (2020); this method is referred to as sparse-DCM. In line with the DCM framework (Friston et al., 2003), sparse-DCM is a state-space model where the state *x*(*t*) satisfies a set of linear differential equations representing the coupling among neural components, and the output model maps the neuronal activity to the measured BOLD signal *y*(*t*) through the hemodynamic response function (HRF):

$$\begin{array}{l} \dot{x}\left(t\right)=Ax\left(t\right)+w\left(t\right),\\ y\left(t\right)=h\left(x\left(t\right);\theta_{h}\right)+e\left(t\right), \end{array}$$ (9)

with *A* representing the effective connectivity matrix, *h*(.) the hemodynamic response that is modeled by the biophysically inspired Balloon-Windkessel model (Friston et al., 2000), and *θ*_*h*_ its parameters. The term *w*(*t*) denotes the stochastic intrinsic brain fluctuations and *e*(*t*) the observation noise. Both are Gaussian variables with zero mean and diagonal covariance matrices *σ*^2^*I*_*n*_ and *R* = diag(*λ*_1_, *λ*_2_, …, *λ*_*n*_), respectively.

To address the computational burden of model inversion when dealing with whole-brain data, Prando et al. (2020) proposed a discretization and linearization of Equation 9 as well as a sparsity-inducing prion on the EC matrix, that is, *A* in Equation 9. This was motivated by the low temporal resolution of fMRI data, which usually ranges from 0.5 to 3 s, and the idea that the hemodynamic response *h*(.) can be modeled as a finite impulse response (FIR) model with input the neuronal state and output the BOLD signal. In our study, to ensure that the length of the input response was large enough to model relevant temporal dependencies, we set the hemodynamic length to 18 samples with a sampling time of 1 s (TR). For each brain parcel *i*, a finite impulse response *h*_*i*_ ∼ 𝒩(*μ*_*h*_, Σ_*h*_) was assigned by deriving *μ*_*h*_ and Σ_*h*_ through a Monte-Carlo sampling of typical responses generated by the nonlinear Balloon-Windkessel model (10,000 samples). The sparsity-inducing prior on the EC estimation was formulated to reduce as much as possible spurious couplings. In particular, each element *a*_*i*_ of matrix *A* was assumed to be a Gaussian variable with zero mean and *γ*_*i*_ variance. The hyperparameter *γ* = [*γ*_1_, *γ*_2_, …, *γ*_*n*^2^_] was estimated through marginal likelihood maximization. Under generic conditions, the maximum likelihood estimate of some *γ*_*i*_-s will be zero such that the Gaussian posterior distribution of their corresponding *a*_*i*_ is concentrated around zero, thus producing a zero MAP estimate. In sparse-DCM, model inversion and parameter optimization are performed by an expectation-maximization (EM) algorithm.

### Metric Description

#### Metrics of time-irreversal asymmetry.

From Gilson et al. (2023), the entropy production rate of a linear stochastic model is

$$\phi =-2\text{tr}\left(\Sigma_{w}^{-1}AS\right),$$ (10)

which is a scale measure that quantifies the degree of irreversibility of the whole process. The local contribution of each node to the global entropy production rate is quantified through the node-irreversibility as ∑_*i*_∣*S*_*ij*_∣. Both metrics are zero in reversible dynamics, indicating no entropy production, while they are greater than zero in irreversible nonequilibrium dynamics.

With the chosen linear state-space model, a nonzero *S* matrix directly influences the distribution of energy within the neuronal state across different frequencies, that is the power spectral density Φ(*ιω*) (Casti et al., 2023):

$$\Phi \left(\iota \omega \right)={\left(\iota \omega I-A\right)}^{-1}\Sigma_{w}{\left(\iota \omega I-A\right)}^{-*},\omega \in \mathbb{R},$$ (11)

with * denoting the conjugate transpose. In particular, the power spectral density of each neuronal state, at a specific frequency *ω*, corresponds to the related diagonal element in Φ(*ιω*), while the off-diagonal elements represent the cross-spectral densities. In the case of time-reversible dynamics, it is expected that the power spectral density will decrease as the frequency *ω* increases. Conversely, for non-time-reversible dynamics, it is anticipated to peak at a frequency greater than zero (Casti et al., 2023).

#### Metastability and synchronization indices.

Metastability and synchronization are indices that describe the phase coherence of a signal ensemble over time (Cabral et al., 2022). The phase coherence of *n* signals is evaluated at each time bin using the Kuramoto order parameter,

$$R\left(t\right)=\frac{1}{n}|\sum_{k=1}^{n}e^{{\iota \phi }_{k}\left(t\right)}|,$$ (12)

where *ϕ*_*k*_(*t*) represents the instantaneous phase of the fMRI signal for node *k*. The value of *R*(*t*) ranges from 0 to 1, where 1 indicates full synchronization and 0 represents randomness—phases uniformly distributed between 0 and 2*π*. The synchronization index is calculated as the mean of *R*(*t*) over time, while the metastability index represents its standard deviation (Shanahan, 2010). The instantaneous phase *ϕ*_*k*_(*t*) of *x*_*k*_(*t*) was computed as the argument of the complex signal *z*_*k*_(*t*) = *x*_*k*_(*t*) + *iH*[*x*_*k*_(*t*)], with *H*[.] denoting the Hilbert transformation. By denoting the module of *z*_*k*_(*t*) with *M*_*k*_(*t*), *x*_*k*_(*t*) can be represented as a rotating vector with phase *ϕ*_*k*_(*t*) and magnitude *M*_*k*_(*t*), that is, *x*_*k*_(*t*) = *M*_*k*_(*t*) cos *ϕ*_*k*_(*t*).

#### DCM as generative model.

After fitting a DCM model to each empirical single-subject rsfMRI recording, we utilized the model to generate synthetic realizations of rsfMRI signals. This procedure was carried out for each dataset using two different approaches: the standard implementation of sparse-DCM proposed in Prando et al. (2020), and a constrained version that enforced the state-space matrix *A* to be symmetric, thereby imposing time-reversibility on the dynamics.

Synthetic data were employed to assess the model’s ability to generate data exhibiting static and dynamic functional connectivity (sFC and dFC, respectively) that resembles the empirical counterparts. To quantify the similarity between sFC, we computed the correlation between the triangular part of empirical and simulated sFC. For dFC, the similarity was determined using the Kolmogorov-Smirnov distance between the triangular parts of empirical and simulated dFC. The calculation of dFC was performed using a sliding window of 50 s with a step of 25 s.

Synthetic data were also used to assess how the synchronization and metastability indices change in data generated by both time-irreversible and time-reversible systems. We aimed to evaluate and compare these indices between the synthetic data and the empirical data, thus gaining insights into the differences arising from the two different dynamics.

## ACKNOWLEDGMENTS

We thank A. Gozzi and L. Coletta from the Functional Neuroimaging Laboratory (Center for Neuroscience and Cognitive Systems @ UniTn, Istituto Italiano di Tecnologia, Rovereto, Italy) for providing the mouse dataset and offering assistance in data curation and preprocessing.

## SUPPORTING INFORMATION

Supporting information for this article is available at https://doi.org/10.1162/netn_a_00381.

## AUTHOR CONTRIBUTIONS

Danilo Benozzo: Conceptualization; Investigation; Methodology; Writing – original draft; Writing – review & editing. Giacomo Baggio: Conceptualization; Methodology; Writing – review & editing. Giorgia Baron: Conceptualization; Data curation; Investigation; Writing – review & editing. Alessandro Chiuso: Conceptualization; Funding acquisition; Supervision; Writing – review & editing. Sandro Zampieri: Conceptualization; Funding acquisition; Supervision; Writing – review & editing. Alessandra Bertoldo: Conceptualization; Funding acquisition; Supervision; Writing – review & editing.

## FUNDING INFORMATION

Department of Information Engineering of the University of Padova (Italy).

## DATA AND MATERIALS AVAILABILITY

The data and code supporting the findings of this study are available in the following GitHub repository: https://github.com/danilobenozzo/dCCov_rsfMRI.git (Benozzo, 2023).

Data were provided in part by the Human Connectome Project, WU-Minn Consortium (principal investigators: David Van Essen and Kamil Ugurbil; 1U54MH091657) funded by the 16 NIH Institutes and Centers that support the NIH Blueprint for Neuroscience Research; and by the McDonnell Center for Systems Neuroscience at Washington University.

## Supplementary Material



## TECHNICAL TERMS

State interaction matrix: The coefficient matrix expressing the rate of change (time derivative) of each state as a linear combination of all states.
Phase space: The space of all possible states of a system.
Jacobian matrix: A matrix containing all first-order partial derivatives of a vector function.
Metastability: A dynamical regime where the system transiently evolves visiting different configurations, i.e., states.
Potential energy: The negative logarithm of the steady-state probability density, it quantifies the surprise at finding the system in any state.
Detailed balance: Condition observed at thermodynamic equilibrium where there is no net flux of transitions between states.
Time arrow: The effect of breaking detailed balance is a non-vanishing rotational flux; its direction defines the arrow of time.
Directed functional hierarchy: Oriented ranking of brain regions based on their functional activities and directed toward the direction of information traveling.

## Supporting Information

https://github.com/danilobenozzo/dCCov_rsfMRI.git
