# Supplementary material for: Analyzing asymmetry in brain hierarchies with a linear state-space model of resting-state fMRI data
Source: Netw Neurosci. 2024 Oct 1;8(3):965–88. doi: 10.1162/netn_a_00381 (PMC11424037; doi:10.1162/netn_a_00381)
Supplement: Supplementary file 1 [file netn-8-3-965-s001.pdf]

# Supplementary Information of: "Analyzing asymmetry in brain hierarchies with a linear state-space model of resting-state fMRI data"

| ID | Brain Area | Abbreviation | Macro Area | Functional Network |
|---|---|---|---|---|
| 1 | Primary motor area | MOp | SM | LCN |
| 2 | Secondary motor area | MOs | PF | LCN/DMNmid |
| 3 | Primary somatosensory area | SSp | SM | LCN |
| 4 | Supplementary somatomotor area | SSs | SM | LCN |
| 5 | Gustatory areas | GU | antL | DMNpostl |
| 6 | Visual areas | VIS | VIS | VIS |
| 7 | Auditory areas | AUD | TEMP | AUD |
| 8 | Anterior cingulate area, dorsal part | ACAd | PF | DMNmid/SAL |
| 9 | Anterior cingulate area, ventral part | ACAv | PF | DMNmid |
| 10 | Prelimbic area | PL | PF | DMNmid |
| 11 | Infralimbic area | ILA | PF | DMNmid |
| 12 | Orbital area | ORB | PF | DMNmid |
| 13 | Agranular insular area, dorsal part | AId | antL | SAL |
| 14 | Agranular insular area, posterior part | AIp | antL | SAL |
| 15 | Agranular insular area, ventral part | AIv | antL | SAL |
| 16 | Retrosplenial area, lateral agranular part | RSPagl | MED | DMNmid |
| 17 | Retrosplenial area, dorsal part | RSPd | MED | DMNmid |
| 18 | Retrosplenial area, ventral part | RSPv | MED | DMNmid |
| 19 | Posterior parietal association areas | PTLp | MED | DMNpostl |
| 20 | Temporal association areas | TEa | TEMP | DMNpostl |
| 21 | Perirhinal area | PERI | TEMP | HP |
| 22 | Ectorhinal area | ECT | TEMP | HP |
| 23 | Visceral area | VISC | antL | DMNpostl |
| 24 | Piriform area | PIR | OLF | not assigned |
| 25 | Ammon's horn | CA | HPF | HP |
| 26 | Dentate gyrus | DG | HPF | HP |
| 27 | Entorhinal area | ENT | HPF | HP |
| 28 | Subiculum | SUB | HPF | HP |
| 29 | Cortical subplate | CTXsp | CTXsp | not assigned |
| 30 | Striatum dorsal region | STRd | STR | DMNsub |
| 31 | Striatum ventral region | STRv | STR | LCNsub |
| 32 | Lateral septal complex | LSX | STR | Basal Forebrain |
| 33 | Striatum-like amygdalr nuclei | sAMY | STR | Basal Forebrain |
| 34 | Pallidum | PAL | PAL | Basal Forebrain |
| 35 | Thalamus, sensory-motor cortex related | DORsm | THAL | THAL |
| 36 | Thalamus, polymodal association cortex related | DORpm | THAL | THAL |
| 37 | Hypothalamus | HY | HY | HY |

Table S1: Mouse brain areas and network information. The same parcellation was applied on both hemispheres. Abbreviations used in the Macro Area column refer to the following areas: somatomotor (SM), medial (MED), temporal (TEMP), visual (VIS), anterolateral (antL), prefrontal (PF) and hippocampal formation (HPF). While abbreviations in the Functional Network column refer to: laterocortical (LCN), subcortical laterocortical (LCNsub), default mode midline (DMNmid), default model posterolateral (DMPpostl), default model subcortical (DMNsub), salience (SAL) and hippocampus (HP).

| ID | Functional network | Brain areas |
|---|---|---|
| 1 | Vis | 1 |
| 2 | Vis | 2/3/4/5/6/7/8/9 |
| 3 | SomMot | 1/2/3 |
| 4 | SomMot | 4 |
| 5 | SomMot | 5/6 |
| 6 | DorsAttn | Post 1 |
| 7 | DorsAttn | Post 2/4/6 |
| 8 | DorsAttn | Post 3/5 |
| 9 | DorsAttn | FEF, PrCv |
| 10 | SalVentAttn | ParOper |
| 11 | SalVentAttn | FrOperIns 1 |
| 12 | SalVentAttn | FrOperIns 2 |
| 13 | SalVentAttn | PFCl |
| 14 | SalVentAttn | Med 1 |
| 15 | SalVentAttn | Med 2 |
| 16 | SalVentAttn | Med 3 |
| 17 | Limbic | OFC |
| 18 | Limbic | TempPole 1 |
| 19 | Limbic | TempPole 2 |
| 20 | Cont | Par |
| 21 | Cont | PFCl |
| 22 | Cont | pCun |
| 23 | Cont | Cing |
| 24 | Default | Temp 1/2, Par 2 |
| 25 | Default | Par 1 |
| 26 | Default | PFC 1 |
| 27 | Default | PFC 2 |
| 28 | Default | PFC 3/5 |
| 29 | Default | PFC 4 |
| 30 | Default | PFC 6 |
| 31 | Default | PFC 7 |
| 32 | Default | pCunPCC 1/2 |
| 63 | Subcortical | Thalamus |
| 64 | Subcortical | Caudate |
| 65 | Subcortical | Putamen |
| 66 | Subcortical | Pallidum |
| 67 | Subcortical | Cerebellum |
| 68 | Subcortical | Hippocampus |

| ID | Functional network | Brain areas |
|---|---|---|
| 33 | Vis | 1 |
| 34 | Vis | 2/3/4/7 |
| 35 | Vis | 5/6/8 |
| 36 | SomMot | 1/2/3 |
| 37 | SomMot | 4 |
| 38 | SomMot | 5/6/7/8 |
| 39 | DorsAttn | Post 1 |
| 40 | DorsAttn | Post 2 |
| 41 | DorsAttn | Post 3/4 |
| 42 | DorsAttn | Post 5 |
| 43 | DorsAttn | FEF, PrCv |
| 44 | SalVentAttn | TempOccPar 1 |
| 45 | SalVentAttn | TempOccPar 2 |
| 46 | SalVentAttn | FrOperIns |
| 47 | SalVentAttn | Med 1/2 |
| 48 | Limbic | OFC |
| 49 | Limbic | TempPole |
| 50 | Cont | Par 1 |
| 51 | Cont | Par 2 |
| 52 | Cont | PFCl 1/2/4 |
| 53 | Cont | PFCl 3 |
| 54 | Cont | Cing, pCun |
| 55 | Cont | PFCmp |
| 56 | Default | Par 1 |
| 57 | Default | Temp 1 |
| 58 | Default | Temp 2/3 |
| 59 | Default | PFCv 1/2 |
| 60 | Default | PFCd PFCm 1/2 |
| 61 | Default | PFCd PFCm 3 |
| 62 | Default | pCunPCC 1/2 |
| 69 | Subcortical | Thalamus |
| 70 | Subcortical | Caudate |
| 71 | Subcortical | Putamen |
| 72 | Subcortical | Pallidum |
| 73 | Subcortical | Hippocampus |
| 74 | Subcortical | Cerebellum |

Table S2: Human brain parcellation and network information (left table, left hemisphere; right table, right hemisphere) This is the result of a consensus clustering algorithm applied to the 100-area cortex Schaefer parcellation and the AAL2 subcortical parcellation.

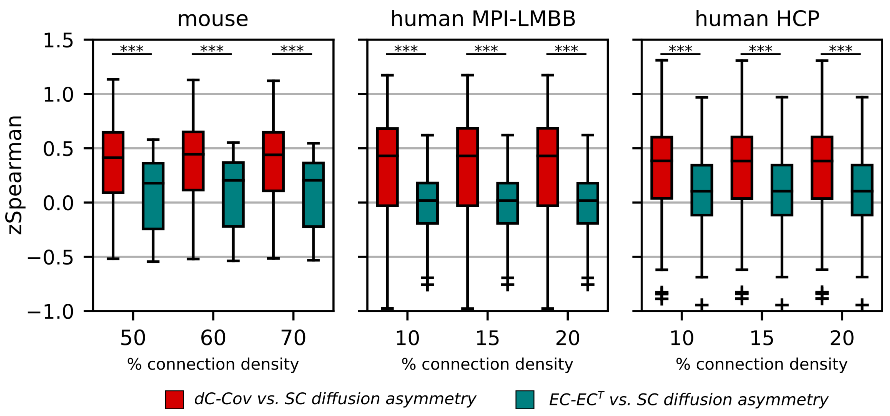


Figure S1: The coupling at the single-subject level with the diffusion efficiency asymmetry, across different SC densities, for each functional dataset. The couplings with diffusion efficiency were computed using both the dC-Cov (red) and $\mathrm{EC} - \mathrm{EC}^\top$ (green). A consistently stronger coupling with structural asymmetry was observed when using dC-Cov. Paired t-test, $p < 0.001$.
